# Supplementary material for: Perceptions of and Preparedness for the Application of Pharmacoeconomics in Practice, among Final Year Bachelor of Pharmacy Students in South Africa: A National Cross-Sectional Study
Source: Pharmacy (Basel). 2023 Mar 14;11(2):54. doi: 10.3390/pharmacy11020054 (PMC10037603; doi:10.3390/pharmacy11020054)
Supplement: Supplementary file 1 [file pharmacy-11-00054-s001.zip › pharmacy-2015406-supplementary.pdf]

Appendix 1: Questionnaire

Perceptions and preparedness of South African final year pharmacy students regarding the practical application of pharmacoeconomics

Consent statement for participation in the study

Thank you for taking time to participate in this study. The questionnaire itself consists of three pages and will take no longer than 15-20 minutes to complete. Please read through this page carefully, before proceeding with the study questions. Do not hesitate to contact the researcher to clarify any uncertainties before completing the survey. Please also take time to rethink your participation in the study.

The aim of the study is to determine the perceptions and preparedness of South African final year Bachelor of Pharmacy students regarding the application of pharmacoeconomics in practice. The objectives of the study are as follows:

- To assess the interpretation of basic pharmacoeconomic concepts and their application in medicines management in South Africa, by final year pharmacy students.
- To ascertain the preparedness of South African final year pharmacy students to apply pharmacoeconomics in practice.
- To determine perceptions of pharmacy students regarding the relevance of pharmacoeconomics in medicines management.
- To determine the need among final year pharmacy students for additional education in pharmacoeconomics.

By completing this questionnaire, you acknowledge that you have heard/read and understand the above aim and objectives of the study, and that they are clear to you. Information obtained in this study may be used in scientific publications nationally and internationally and will subsequently be available electronically. Results of this research will be used for scientific purposes. By completing the questionnaire, you consent to this since your name is not provided anywhere in this survey and is therefore completed anonymously. Participation in this project is completely voluntary, and you were not pressurised, in any way, to participate.

This project has been granted ethical approval by the Sefako Makgatho Science University Research Ethics Committee (SMUREC). The SMUREC Ethics Reference Number is SMUREC/P/97/2018: PG.

Demographic information

Please tick the appropriate box to the following:

x

**Gender:**		Male		Female		Other, please specify:

**Race:**		African		Asian		Coloured		Indian		White
		Other, please specify:								

**Age:**		Years		Months	**or DOB**	Y	Y	Y	Y	-	M	M	-	D	D		

**Please indicate at which academic institution you are currently enrolled**			
Nelson Mandela University (NMU)		University of KwaZulu-Natal (UKZN)	
North-West University (NWU)		University of Limpopo (UL)	
Rhodes University (RU)		University of the Western Cape (UWC)	
Sefako Makgatho Health Sciences University (SMU)		University of the Witwatersrand (WITS)	
Tshwane University of Technology (TUT)			
**Do you hold any other degree(s)?**			
a. No			
b. Yes, please specify:			
**Please indicate in which sector you intend on completing your internship**			
a. Academic institution		d. Public institutional pharmacy	
b. Community pharmacy		e. Manufacturing pharmacy	
c. Private institutional pharmacy		f. Don't know	

Pharmacoeconomics education during BPharm degree programme

Please answer the following question:

1.	**What is the name of the subject/module/course under which pharmacoeconomics was offered during your BPharm degree programme?**

Please choose one answer to each of the following questions:

2.	**At which level of the BPharm programme was pharmacoeconomics offered?**			
	a. 3rd Year		c. Both 3rd and 4th year	
	b. 4th Year			
3.	**Was pharmacoeconomics offered as a mandatory or elective subject/module/course during your BPharm degree programme?**			
	a. Mandatory subject/module/course		c. Don't know	
	b. Elective subject/module/course		d. Other, please specify:	
4.	**Was your knowledge on pharmacoeconomics tested during your BPharm degree programme?**			
	a. No			
	b. Yes, please specify how it was tested:			

Please answer the following question:

5.	**How was pharmacoeconomics taught during your BPharm degree programme and what is the estimated amount of time (clock hours) spent on teaching?** (may choose more than one option)				
	a. Lectures	**Standard lecture duration (minutes)**		**Total number of lectures spent on teaching pharmacoeconomics**	
	b. Practical work	**Standard duration of practical work (minutes)**		**Total number of practical work sessions spent on pharmacoeconomics**	
	c. Tutorials	**Standard tutorial duration (minutes)**		**Total number of tutorials spent on teaching pharmacoeconomics**	
	d. Workshops	**Standard lecture duration (minutes)**		**Total number of lectures spent on teaching pharmacoeconomics**	
	e. Other, please specify:				

Pharmacoeconomic concepts

Please choose <u>one</u> answer to each of the following statements on the **scope of pharmacoeconomics**:

6.	**Examines and calculates the costs of medicines and treatments <u>only</u>**	True	False	Don't know
	**Compares different pharmaceutical interventions to each other**	True	False	Don't know
	**Evaluates and measures the costs <u>and</u> benefits of drug therapy**	True	False	Don't know
	**Involves a combination of health economics and clinical outcomes**	True	False	Don't know
	**Measures the impact of the costs of medicines on medicine budgets**	True	False	Don't know

Please indicate your level of understanding of the following pharmacoeconomic concepts by ticking the most appropriate answer:

		I have no understanding	I have no understanding but would like to	I have some understanding	I understand this term and can explain it to others	N/A
7.	**Cost-minimisation analysis (CMA)**					
	**Cost-benefit analysis (CBA)**					
	**Cost-effectiveness analysis (CEA)**					
	**Cost-utility analysis (CUA)**					
	**Incremental cost-effectiveness ratio (ICER)**					
	**Sensitivity analysis**					
	**Opportunity costs**					
	**Discounting**					
	**Quality adjusted life year (QALY)**					
	**Disability adjusted life year (DALY)**					

The input (costs) for the following pharmacoeconomic analyses are all measured in **monetary units (Rands)**. Please match an applicable <u>outcome measurement unit</u> for each analysis, by choosing the appropriate number:

8.

Method of pharmacoeconomic evaluation	No.
**Cost-minimisation analysis (CMA)**	
**Cost-benefit analysis (CBA)**	
**Cost-effectiveness analysis (CEA)**	
**Cost-utility analysis (CUA)**	

No.	How outcomes (health benefits) are measured
1	Health benefits across therapies are valued in similar units, depending on individual preference
2	Health benefits assumed to be equivalent and can take any form (e.g. number of life years saved, reduction in cholesterol levels)
3	Measured in similar or different units and always valued in monetary units (e.g. amount willing to pay to prevent injury)
4	Health benefits across therapies are measured in similar natural health units

Preparedness for application of pharmacoeconomics in practice

Please indicate your level of agreement with the following statements by ticking the most appropriate answer:

9.	**I received enough exposure to pharmacoeconomics during my BPharm degree programme to understand the basic pharmacoeconomic principles and concepts**	Strongly disagree	Disagree	No opinion	Agree	Strongly agree
10.	**I am adequately prepared to apply fundamental pharmacoeconomic concepts in practice to conduct a pharmacoeconomic analysis**	Strongly disagree	Disagree	No opinion	Agree	Strongly agree
11.	**I can interpret the results of a pharmacoeconomic analysis/study and make a decision based on my interpretation**	Strongly disagree	Disagree	No opinion	Agree	Strongly agree
12.	**I know where to find more information on pharmacoeconomic concepts**	Strongly disagree	Disagree	No opinion	Agree	Strongly agree
13.	**I find pharmacoeconomics interesting and enjoyable**	Strongly disagree	Disagree	No opinion	Agree	Strongly agree

Please indicate how competent you consider yourself to perform the following pharmacoeconomic analyses, by ticking the most appropriate answer:

14.	**Cost-minimisation analysis (CMA)**	Very incompetent	Incompetent	No opinion	Competent	Very competent
	**Cost-benefit analysis (CBA)**	Very incompetent	Incompetent	No opinion	Competent	Very competent
	**Cost-effectiveness analysis (CEA)**	Very incompetent	Incompetent	No opinion	Competent	Very competent
	**Cost-utility analysis (CUA)**	Very incompetent	Incompetent	No opinion	Competent	Very competent

Please indicate how often you expect to perform the following pharmacoeconomic analyses in practice:

15.	**Cost-minimisation analysis (CMA)**	Never	Rarely	Often
	**Cost-benefit analysis (CBA)**	Never	Rarely	Often
	**Cost-effectiveness analysis (CEA)**	Never	Rarely	Often
	**Cost-utility analysis (CUA)**	Never	Rarely	Often

Relevance of pharmacoeconomics in practice

Please answer the following question:

16.	**Which professionals do you think, should perform pharmacoeconomic analyses for use in practice?** (may choose more than one option)			
	a. Health economists		f. Medical practitioners	
	b. Economists		g. Epidemiologists	
	c. Accountants		h. Demographers	
	d. Pharmacists		i. People with a mathematical background	
	e. Nursing practitioners		j. Mathematical modellers	

Please indicate your level of agreement with the following statements on application of pharmacoeconomics in South Africa by ticking the most appropriate answer:

17.	**Ensures optimal use of medicine budgets across the South African public health sector**	Strongly disagree	Disagree	No opinion	Agree	Strongly agree
18.	**Should form an integral part of the South African National Health Insurance system**	Strongly disagree	Disagree	No opinion	Agree	Strongly agree
19.	**Will improve access to medicines**	Strongly disagree	Disagree	No opinion	Agree	Strongly agree
20.	**An important skill which South African pharmacists should possess**	Strongly disagree	Disagree	No opinion	Agree	Strongly agree
21.	**Improves medicine-related decisions in the South African healthcare system**	Strongly disagree	Disagree	No opinion	Agree	Strongly agree

Please indicate your level of agreement about the instances where pharmacoeconomics is used in South Africa:

22.	**Planning of production and sales of medicines**	Strongly disagree	Disagree	No opinion	Agree	Strongly agree
23.	**Pricing of medicines**	Strongly disagree	Disagree	No opinion	Agree	Strongly agree
24.	**Clinical decision-making at an individual patient level, in case of special motivation for medicine not available on the Essential Medicines List (EML), formulary or Standard Treatment Guidelines (STGs)**	Strongly disagree	Disagree	No opinion	Agree	Strongly agree
25.	**Registration of new medicines with the South African Health Products Regulatory Authority (SAHPRA)**	Strongly disagree	Disagree	No opinion	Agree	Strongly agree
26.	**Inclusion of medicines in medicine formularies (e.g. EML, medical aid formularies, STGs)**	Strongly disagree	Disagree	No opinion	Agree	Strongly agree

Please choose one answer to the following question:

27.	**In your opinion, to which South African health sector is application of pharmacoeconomics relevant?**			
	a. Private sector only (e.g. Medical aid formularies)		c. Both private and public sector	
	b. Public sector only (e.g. Essential Medicines List, Standard Treatment Guidelines)		d. Don't know	

Future education in pharmacoeconomics

Please indicate your level of agreement with the following statements by ticking the most appropriate answer:

28.	**Future education in pharmacoeconomics is essential for my role as pharmacist**	Strongly disagree	Disagree	No opinion	Agree	Strongly agree
29.	**I would have liked to receive more pharmacoeconomics training at an undergraduate level**	Strongly disagree	Disagree	No opinion	Agree	Strongly agree

Please choose one answer to the following questions:

30.	**Would you like to acquire additional knowledge on pharmacoeconomics in future?**	
	a. Yes, please proceed to Question 31	
	b. No, I would not like to acquire additional knowledge on pharmacoeconomics	

31.	**How would you like to acquire additional knowledge regarding pharmacoeconomics?**			
	a. Continuous professional development (CPD) programme		c. Postgraduate studies	
	b. Self-study			

**Provide any additional comments or thoughts on pharmacoeconomics and its application in the practice of pharmacy**

Thank you for your time and participation!
